# Comparing travel behaviour characteristics and correlates between large and small Kenyan cities (Nairobi versus Kisumu)

**DOI:** 10.1016/j.jtrangeo.2023.103625

**Published:** 2023-06

**Authors:** Lambed Tatah, Louise Foley, Tolu Oni, Matthew Pearce, Charles Lwanga, Vincent Were, Felix Assah, Yves Wasnyo, Ebele Mogo, Gabriel Okello, Stephen Mogere, Charles Obonyo, James Woodcock

**Affiliations:** a MRC Epidemiology Unit, University of Cambridge, Cambridge, United Kingdom; b Center for Global Health Research, Kenya Medical Research Institute (KEMRI), P.O. Box 1578, Kisumu 40100, Kenya; c Health of Populations in Transition (HoPiT) Research Group, Faculty of Medicine and Biomedical Sciences, The University of Yaoundé I, Yaoundé, Cameroon; d Institute of Sustainability Leadership, University of Cambridge, Cambridge, United Kingdom; e Japan International Cooperation Agency (JICA), Britam Tower 22nd & 23rd Flrs, Upper Hill Road, P.O. Box 50572-00200, Nairobi, Kenya

**Keywords:** Travel behaviour, Urban transport, Nairobi, Kisumu, Kenya

## Abstract

Understanding urban travel behaviour is crucial for planning healthy and sustainable cities. Africa is urbanising at one of the fastest rates in the world and urgently needs this knowledge. However, the data and literature on urban travel behaviour, their correlates, and their variation across African cities are limited. We aimed to describe and compare travel behaviour characteristics and correlates of two Kenyan cities (Nairobi and Kisumu). We analysed data from 16,793 participants (10,000 households) in a 2013 Japan International Cooperation Agency (JICA) household travel survey in Nairobi and 5790 participants (2760 households) in a 2016 Institute for Transportation and Development Policy (ITDP) household travel survey in Kisumu. We used the Heckman selection model to explore correlations of travel duration by trip mode. The proportion of individuals reporting no trips was far higher in Kisumu (47% vs 5%). For participants with trips, the mean number [lower - upper quartiles] of daily trips was similar (Kisumu (2.2 [2–2] versus 2.4 [2–2] trips), but total daily travel durations were lower in Kisumu (65 [30–80] versus 116 [60–150] minutes). Walking was the most common trip mode in both cities (61% in Kisumu and 42% in Nairobi), followed by motorcycles (17%), matatus (minibuses) (11%), and cars (5%) in Kisumu; and matatus (28%), cars (12%) and buses (12%) in Nairobi. In both cities, females were less likely to make trips, and when they did, they travelled for shorter durations; people living in households with higher incomes were more likely to travel and did so for longer durations. Gender, income, occupation, and household vehicle ownership were associated differently with trip making, use of transport modes and daily travel times in cities. These findings illustrate marked differences in reported travel behaviour characteristics and correlates within the same country, indicating setting-dependent influences on travel behaviour. More sub-national data collection and harmonisation are needed to build a more nuanced understanding of patterns and drivers of travel behaviour in African cities.

## 1 Introduction

Understanding urban travel behaviour is crucial for planning healthy and sustainable cities. It helps us estimate the population risk of road traffic injuries, the physical activity resulting from active travel, and transport-related air pollution and greenhouse gas emissions (Dhondt et al., 2013; Griffiths et al., 2000). Measuring these indicators is an effective way of planning healthy and sustainable cities, for what gets measured gets done (Giles-Corti et al., 2022). The harms resulting from poor transport planning need urgent tackling because they contribute significantly to planetary health and the global disease burden. For example, road traffic injuries account for 1.35 million deaths per year globally, ranking among the top ten causes of mortality in the general population and the highest cause of death in people aged between 15 and 29 years (World Health Organisation, 2018). Physical inactivity and air pollution are estimated to be responsible for 4 million (Strain et al., 2020a) and 7 million deaths per year, respectively (World Health Assembly, 2015), mainly through non-communicable diseases (NCDs), which are responsible for 71% of global mortality (Bennett et al., 2018).

Knowledge of urban travel behaviour is limited in low- and middle-income countries (LMICs), especially in Africa (Foley et al., 2021; Salon and Gulyani, 2019). It is often said that most people walk in African cities, but how this varies and how much of urban travelling is done by cycling, motorcycling, or informal minibuses is simply unclear—people generalise across the large continent. In addition to the knowledge limitation, transport authorities are fragmented and underfunded (Kumar and Barrett, 2008); these factors lead to inadequate urban transport planning with visible consequences. A vast majority (93%) of road traffic fatalities occur in LMICs, mostly in sub-Saharan African countries (Vissoci et al., 2017), and 77% of global NCD deaths occur in LMICs (Bennett et al., 2018). Non-health problems associated with inadequate urban transport planning in these settings include high traffic congestion and associated long and highly unpredictable trip and daily travel durations. For example, in Nairobi, Kenya, traffic models estimate that vehicle speeds are respectively 8.3 km/h and 7.6 km/h during the morning and evening peaks (Gonzales et al., 2011). In Dar es Salaam, Tanzania, daytime average speeds are 8–15 km/h (Melbye et al., 2015), and trips can take anything from 30 min to 3 h depending on the traffic conditions in the city (Andreasen and Møller-Jensen, 2017). In addition, there are significant gender and socioeconomic inequities in mobility (Bryceson et al., 2003; Foley et al., 2021; Salon and Gulyani, 2010; Venter et al., 2007) and challenges posed by the dominant informal public transport system (Kamuhanda and Schmidt, 2009).

The gaps in the literature on African urban travel behaviour stem primarily from the lack of routine surveys and the inadequate exploitation of the few available data. The scant literature provides insights into transport challenges in some thematic areas, such as gender and socioeconomic inequities in mobility, but these are generally not based on representative survey data. Moreover, the available literature (Abane, 2011; Behrens and Behrens, 2004; Gebeyehu and Takano, 2007; Salon and Aligula, 2012) mainly reports summary statistics relating to vehicle ownership, transport mode shares and expenditure on transport, which are limited in terms of travel time, who is travelling and for what purpose. Crucially, there is a lack of comprehensive explanatory analyses to show the correlates of these variables and how these vary across African cities, especially large and small cities.

Comparing travel behaviours among cities provides not only valuable information on the risk of road traffic injuries, air pollution, and transport-related physical activity but also yields transferable lessons which cities at different growth stages can use to tackle urban transport challenges. For example, if the factors favouring active travel in one city are modifiable and applicable in another. This information is also crucial for national governments, which need to design policies on urban transport that account for the different needs of cities at different growth stages. Given that Africa is urbanising at one of the fastest rates in the world (United Nations, 2019) and in a largely unplanned manner that gives rise to major informal settlements (World Bank, 2018), there is an urgent need to enable healthy and sustainable urban transport planning to promote urban population health and wellbeing through shared learning.

This study compares travel behaviour characteristics and correlates between two Kenyan cities: Nairobi (the capital city) and Kisumu (the third-largest city in Kenya). Travel behaviour characteristics include trip number, mode, duration, and purpose (Van Acker et al., 2010). The study addresses two specific questions: What are the household, individual, and travel behaviour characteristics and correlates in Nairobi and Kisumu? How do travel behaviour characteristics and correlates differ between the two cities?

## 2 Methods

### 2.1 Study design

We used two cross-sectional travel surveys from two cities in Kenya (the 2013 Japan International Cooperation Agency (JICA) household travel survey in Nairobi (Nairobi City Council, 2014) and the 2016 Institute for Transportation and Development Policy (ITDP) household travel survey in Kisumu (Institute for Transportation and Development Policy, 2020)) to describe and compare travel behaviour characteristics and correlates between Nairobi and Kisumu. To ensure the inclusion of essential survey elements, we reported the study following the Strengthening the Reporting of Observational Studies in Epidemiology (STROBE) guidelines (Cuschieri, 2019).

### 2.2 Settings

**Fig. 1:**
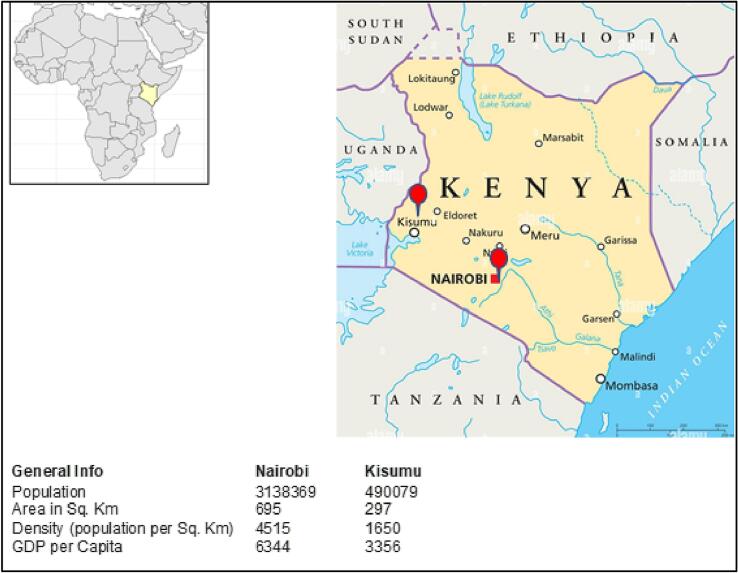

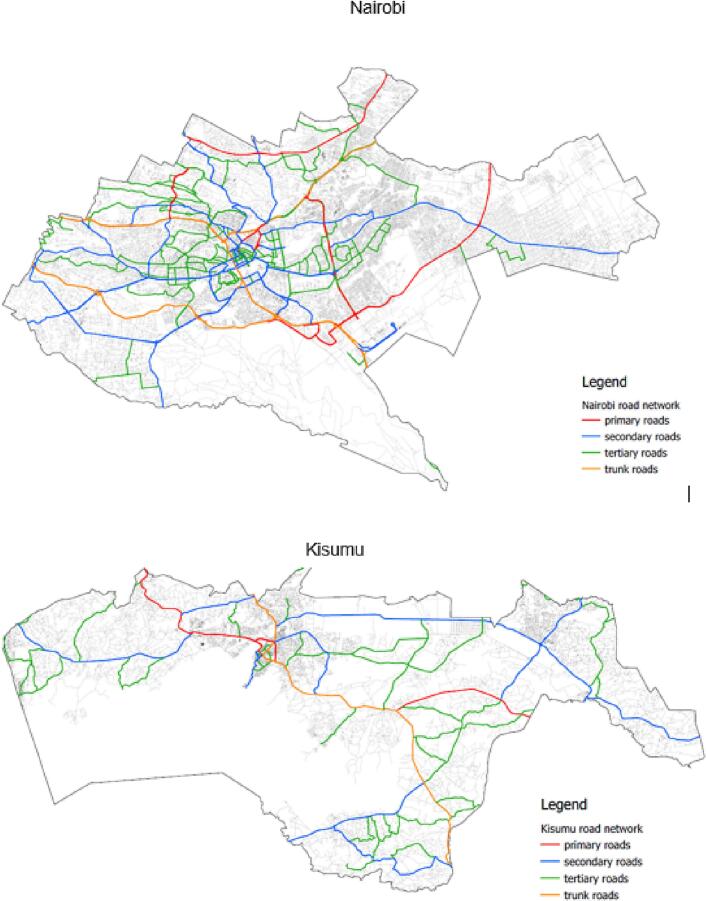
a: Map of Kenya showing Nairobi and Kisumu and city characteristics (numbers scaled to 2013 projections; per capita GDP estimated in Kenyan Shillings (KES)). b: Maps of road networks and density in Kisumu and Nairobi, Kenya (drawn from the Open Street Map).

The study is based in two Kenyan cities: Nairobi and Kisumu (Fig. 1a and b). Like most African cities, Nairobi and Kisumu are experiencing rapid transportation changes, including rapid vehicle ownership growth. According to the Kenyan National Bureau of Statistics (KNBS), the number of cars, motorcycles, and all vehicles registered in 2012 were 644,805, 610,056 and 1,789,789, with a five-year growth (2008–2012) of 9%, 30% and 12%, respectively. The average number of vehicles per capita was 0.076 in 2019, up from 0.024 in 2010.

Nairobi, the nation's capital, is densely populated and located in the country's central highlands. Based on the 2009 Kenyan household census, the city had an estimated population of 3.1 million people and a density of 4515 people/km^2^ in 2013. As Kenya's largest city, it attracts many people from other urban and rural areas in search of better economic and livelihood opportunities. This has led to rapid and uncontrolled urban growth, congestion of streets and residential areas, and proliferation of slums, with 60%–70% of Nairobi's population currently living in slums (African Population and Health Research Center (APHRC), 2018). Much of Nairobi's population lives on the outskirts and commutes to the city centre daily (NASA Earth Observatory, 2016). Main public transport modes include matatus (minibuses), motorcycles (bodaboda), and walking. Private cars are aspirational as people try to avoid the discomfort of using matatus, and even though personal car use remains low, traffic congestion and associated harms are increasing. Walking is a common aspect of journeys, both as the main mode and as part of other journeys, weaving through busy roads, dodging open drains, potholes, street vendors and speeding vehicles.

Kisumu is Kenya's third most populous city but far smaller than Nairobi's population. It is located in the west and borders Lake Victoria. Based on the 2009 Kenyan household census, Kisumu had an estimated population of 490,079 and a density of 297 people/km^2^ in 2013. Like many African cities, Kisumu is starting to experience mobility challenges characterised by increasing car traffic, inefficient public transport, inadequate walking and cycling facilities, and poor traffic and parking management. These challenges contribute to air and noise pollution, congestion, and safety concerns, impacting the city's attractiveness and liveability.

### 2.3 Participants

Participants in both surveys were members of selected households aged five years and above. Individuals were considered household members if they were normal residents of the households or visitors who had spent the previous night in the household. Households were selected from established sampling frames as described below.

### 2.4 Data

The two main data sources for this study were collected by JICA in 2013 in Nairobi and ITDP in 2016 in Kisumu. A third data source, by the World Bank (2013 Kenya - State of the City Baseline Survey), was used to compare key indicators in the JICA and ITDP data.

JICA surveyed a total of 10,000 households and 16,580 individuals in Nairobi in 2013 (Nairobi City Council, 2014). The JICA team used a small zone system to stratify the city into 106 zones to obtain this sample. The team based their household sampling rate on the 2009 population housing census. With 985,016 households in Nairobi, the estimated sampling rate for 10,000 households was 1.02% per zone. The survey was conducted in person in the selected households, and questions covered variables related to household income and assets, as well as age, education, and travel information for all household members aged five years and above.

ITDP surveyed 2760 households and 5970 individuals in Kisumu in 2016 (Institute for Transportation and Development Policy, 2020). The team based their household sampling on the 2009 population housing census. Their sampling strategy also comprised a small zone system that stratified the city into 104 zones. The survey was conducted in person in the selected households, and questions covered variables related to household income and assets, as well as age, education, and travel information for all household members above the age of five years.

Both surveys included household questions on household composition, income, and vehicle ownership; individual questions on demographics, income and occupation; and 24-h trip diaries covering the number of trips taken, trip stages, modes, purposes and costs. Additional questions in Kisumu covered perceptions of the travel environment and opinions to improve travel in the city.

To account for differences that potentially stemmed from comparing datasets collected by two different institutions at different locations and times, we compared commute characteristics from the two analysis datasets with those from the 2013 World Bank survey in Kenya. The World Bank survey collected baseline data on the state of the cities in Kenya, including data on usual commutes to school and work.

### 2.5 Variables

Predictor variables included household and individual characteristics. Household characteristics included household size (number of individuals in each household), household monthly income, and household vehicle ownership. Individual characteristics included age, age group (5–14, 15–24, 25–34, 35–44, 45–54, 55–64, 65+ years), sex, monthly income, and occupation (no work, employed, housekeeper, student, other). Individual monthly income was grouped into three categories to reflect the minimum monthly wage in Kenya, which was <10,000 KES (Kenya Shilling) between 2013 and 2016 (<10,000 KES, 10000–30,000 KES and > 30,000 KSH) (Trading Economics, 2021). One Kenyan Shilling was exchanged for an average of $0.01 between 2013 and 2016.

Outcome variables included individual trip characteristics. A trip was defined as one journey undertaken by a participant from origin to destination for a single purpose during the reference day. ‘No trip’ was defined as not making a trip on the reference survey day. A trip could have multiple stages, denoting the different stops to change transport modes during the trip. A trip mode was defined as the largest transport mode used during the trip, while the stage mode was the transport mode used in each trip stage. Transport modes included walking, cycling, motorcycle, tricycle, car, taxi, matatu (minibus), bus, truck, and train. Matatu refers to privately owned small-capacity (14–30) minibuses used as shared taxis, while a bus refers to a publicly owned larger-capacity vehicle running on fixed routes and schedules. Transport modes were grouped into non-motorised transport (NMT) (walking and cycling) and motorised transport (MT) (motorcycle, tricycle, car, taxi, matatu, bus, train). Motorised transport was further grouped into private MT (car, private motorcycle, private tricycle) and public MT (commercial motorcycle or tricycle, taxi, matatu, and bus); and public MT was also grouped into formal public MT (bus) and informal public MT (commercial motorcycle or tricycle, taxi, and matatu). The trip duration was the sum of stage durations within one trip, and the daily travel duration was the sum of all trip durations for trips travelled by an individual. Trip purposes included travel for work, school, recreation, shopping, personal business, return home and others.

### 2.6 Statistical analysis

For our exploratory analysis, we used a combination of summary statistics and plots to check for missingness, normality, zero inflation, and near-zero variance in data variables. With 3% and 5% of observations with missing data points in the Nairobi and Kisumu travel datasets, respectively, we simply excluded missing records from each relevant analysis. Our variables of interest did not show near-zero variance (categorical variables). Total daily travel duration and daily travel duration by mode were right-skewed; these were log-transformed.

We summarised and compared household and participant characteristics for each site. We also summarised and compared travel behaviour characteristics (trip frequency and time, use of different transport modes, mean (lower - upper quartile) travel times by mode and trip purposes) by age group and sex for each study site. We used the mean (lower - upper quartile) to summarise variables with higher proportions of zeros, which could otherwise be all zeros if the median (interquartile range) was used. Our descriptive statistics were weighted using postratification weights derived from sex and age distribution. These demographic distributions were obtained from the 2014 population demographic data, which was a projection from the 2009 national population census.

We used a Toby type 2 Heckman selection modelling framework to evaluate correlates of trip making, use of different transport modes (NMT, informal PT and private MT), daily travel duration and travel durations in different transport modes. Our model choice was informed by the high frequencies of unobserved zeros in the data, especially for Kisumu. The total zeros were far more than the expected zeros in the case of a corner solution, suggesting unobserved zeros, which could result from soft refusal (participants reporting travel to avoid answering long survey questions). The model framework comprised two equations. The first equation was a probit model that evaluated the likelihood of making a trip and using a transport mode. The probit model also generated the inverse mills ratios (imr) which were used to parameterise the second equation. The second equation was an ordinary least squares (OLS) model that evaluated the correlates of the log-transformed daily travel durations. In addition, we used a Poisson regression to predict correlates of trip frequencies. All analyses were performed in the R software, notably using the “sampleSelection” package (version 1.2–12) for our modelling.

We reported the outputs of the probit model as average marginal effects (AME), indicating the change in probability for any unit change in predictor variables. The outputs of the Poisson regression were reported as rate ratios. We reported the outputs of the OLS as per cent change in the daily travel time for a unit change in the predictors since the dependent variable was log-transformed. We reported the point estimates and their 95% confidence intervals for each dependent variable.

## 3 Results

### 3.1 Characteristics of the study sample

We analysed data from 10,000 households and 16,794 participants in Nairobi and 2760 households with 5790 participants in Kisumu. Household and participant characteristics are summarised in Table 1. Compared to Nairobi, a higher proportion of households in Kisumu registered no response to household income (32% vs 1%). A higher proportion of households in Kisumu owned bicycles (15% vs 10%, *p* < 0.001), while ownership of all forms of motorised vehicles was lower in Kisumu. Participants from Kisumu were younger and comprised more females, students, and people with no employment.

**Table 1:** Characteristics of households and participants surveyed in Nairobi (2013) and Kisumu (2016), Kenya.

	Nairobi unweighted	Nairobi weighted	Kisumu unweighted	Kisumu weighted
Household characteristics	*N* = 10,000		*N* = 2760	
Median household size [IQR]	2.6 [1–3]	..	2.1 [1–3]	..
Household income in KES/month (%)				
<10,000	2000(20)	..	1049(38)	..
10,000–30,000	4100(41)	..	607(22)	..
>30,000	3700(37)	..	221(8)	..
No response	100(1)	..	883(32)	..
% Households with vehicles (number per 100 households)				
Bicycle	10 (12)	..	15 (17)	..
Motorcycle	5 (6)	..	11 (13)	..
Car	19 (27)	..	8 (12)	..
Motorised vehicle	24 (35)	..	12 (16)	..
Any vehicle	30 (47)	..	25 (33)	..

Participant characteristics	*N* = 16,794	N = 16,794	*N* = 5790	N = 5790
Average age (SD)	30 (11)	27 (13)	27 (15)	25 (18)
Age group in years (n, %)				
5–14	1276 (8)	3398 (20)	1161 (20)	1870 (32)
15–24	3418 (20)	4585 (27)	1475 (26)	1557 (27)
25–34	7276 (43)	4827 (29)	1743 (30)	1000 (17)
35–44	3268 (20)	2308 (14)	800 (14)	530 (9)
45–54	1183 (7)	1065 (6)	306 (5)	376 (7)
55–64	285 (2)	395 (2)	197 (3)	233 (4)
65+	88 (1)	216 (1)	108 (2)	224 (4)
Female (n, %)	8456 (50)	8181 (49)	2612 (45)	2966 (51)
Occupation (n, %)				
No work	959 (6)	983 (6)	590 (10)	458 (8)
Student	2572 (15)	4929 (29)	1551 (27)	1981 (34)
Employed	12,009 (72)	9802 (58)	2779 (48)	2311 (40)
Homemaker	1071 (6)	879 (5)	448 (8)	358 (6)
Other	183 (1)	201 (1)	422 (7)	682 (12)
Participants in household income groups in KES/month (n, %)				
<10,000	5328 (32)	6042 (36)	1622 (28)	13,156 (23)
10,000–30,000	5586 (33)	4499 (27)	666 (12)	498 (7)
>30,000	3634 (22)	2860 (17)	125 (2)	108 (2)
No response	2246 (13)	3393 (20)	3377 (58)	3867 (67)

KES = Kenyan Shilling; SD = standard deviation; IQR = interquartile range.

### 3.2 Travel behaviour characteristics

The trip characteristics in each study site are summarised in Table 2. Compared to Nairobi, the proportion of participants reporting no trip in the 24-h trip diary period was far higher in Kisumu (47% vs 5%). The overall mean number of trips per survey participant was lower in Kisumu (1.2; IQR:0–2 vs 2.3; IQR:2–2; *p* < 0.001), but when participants with no trips were excluded, the mean number of trips per participant with trips was similar in both cities (2.2; IQR:2–2 in Kisumu vs 2.4; IQR:2–2 in Nairobi; p < 0.001). The mean duration per trip, mean total travel time per survey participant (including participants with no trips) and total travel time per survey participant (excluding participants with no trips) in Kisumu were half of those in Nairobi.

**Table 2:** Characteristics of trips reported in the 24-h trip diary in Nairobi (2013) and Kisumu (2016), Kenya.

Trip characteristics	Nairobi	Kisumu
Proportion of participants reporting no travel (%)	5	47
Total number of trips	37,779	6892
Mean [IQR] number of trips per survey participant	2.3 [2–2]	1.2 [0–2]
Mean [IQR] number of trips per participant with trips	2.4 [2–2]	2.2 [2–2]
Mean [IQR] number of stages per trip	2.1 [1–3]	1.3 [1–1]
Mean [IQR] trip time in minutes per trip	49 [30–60]	24 [10−30]
Mean [IQR] total travel time in minutes per survey participant	110 [60–150]	50 [10–70]
Mean [IQR] total travel time in minutes per participant with trips	116 [60–150]	65 [30–80]

IQR = Interquartile range.

Walking was the most common trip mode in both cities for both sexes, with most walking trips undertaken by females (Table 3). Compared to Nairobi, the proportion of trips on bi/tri/motorcycles was higher, while the proportion of trips in cars, matatus or buses was lower in Kisumu. In both cities, most bi/motorcycle, car, and bus trips were undertaken by males, while matatu trips were evenly distributed between sexes.

**Table 3:** Trip mode and purpose share and duration by sex in Nairobi (2013) and Kisumu (2016), Kenya.

	Nairobi						Kisumu					
	Mode share (%)			Mean duration (IQR) in minutes			Mode share (%)			Mean duration (IQR) in minutes		
	Both sexes	Male	Female	Overall	Male	Female	Both sexes	Male	Female	Overall	Male	Female
	*N* = 37,779	*N* = 19,475	*N* = 18,322				6892	3447	3445			
Main trip mode												
Walk	41.7	36.6	47.2	33(15–30)	34(15–30)	32(15–30)	61.1	54.6	67.5	20(10–26)	20(10–26)	20(10–28)
Bicycle	1.4	2.4	0.4	43(20–60)	42(20–60)	48(30–60)	4.6	7.2	1.9	20(10−30)	20(10–28)	21(10–30)
Motorcycle	3.4	3.5	3.3	38(25–45)	42(25–60)	34(20–40)	2.7	2.4	3	27(15–35)	28(15–35)	27(18–35)
Tricycle	1	0.9	1.2	49(30–60)	47(30–60)	50(30–60)	14.9	16.9	12.9	19(10–25)	18(10−20)	21(10–25)
Car	11.7	15.3	7.9	54(30–60)	56(30–60)	51(30–60)	4.8	6.7	2.9	24(10–30)	25(10–30)	23(15–30)
Matatu	27.7	27.7	27.8	60(30–70)	62(30–75)	57(30–60)	10.5	10.4	10.6	41(25–50)	42(25–50)	40(22–50)
Bus	11.8	12.4	11.3	59(30–70)	59(30–75)	58(30–70)	1	1.3	0.7	42(15–45)	52(25–45)	22(15–30)
Other	1.1	1.2	1	49(30–60)	57(30–60)	39(20–45)	0.5	0.5	0.5	47(10–50)	80(24–95)	12(8–10)
Overall	100	100	100	46(25–60)	49(30–60)	44(20–60)	100	100	100	23(10–30)	23(10–30)	22(10–30)

Grouped modes												
NMT	43.1	39	47.5	33(15–30)	34(15–30)	32(15–30)	65.6	61.9	69.4	20(10–28)	20(10–28)	20(10–30)
Informal PT	32.1	32	32.2	57(30–60)	59(30–70)	55(30–60)	23.3	22.4	24.3	30(15–37)	30(15–39)	30(15–35)
Formal PT	12.1	12.7	11.4	59(30–75)	60(30–75)	58(30–72)	1	1.3	0.7	42(15–45)	52(25–45)	22(15–30)
Private MT	11.8	15.4	8	54(30–60)	56(30–60)	51(30–60)	9.6	14	5.2	22(10–30)	21(10–25)	23(15–30)
Other	0.9	1	0.9	43(30–45)	53(30–60)	32(20−30)	0.5	0.5	0.5	47(10–50)	80(24–95)	12(8–10)
Overall	100	100	100	46(25–60)	49(30–60)	44(20–60)	100	100	100	23(10–30)	23(10–30)	22(10–30)

Trip purpose												
Work	24.9	29.1	20.5	50(30–60)	52(30–60)	47(30–60)	12.7	16.3	9	28(10–30)	28(10–30)	28(10–30)
Education	15.3	14.7	15.9	37(20–45)	37(20–45)	37(20–45)	17.5	18.7	16.2	19(10–25)	19(10–25)	19(10–30)
Personal	6.2	6	6.4	43(20–60)	44(20–60)	43(20–60)	12.2	12.4	12.1	26(10–30)	25(10–30)	27(10–35)
Recreation	2.1	2.1	2.1	49(20–60)	48(20–60)	51(20–60)	1.3	1.5	1.1	23(10–30)	21(10–30)	25(10–30)
Shopping	5	2.5	7.7	35(15–45)	37(15–50)	35(15–40)	8.6	3.7	13.5	16(5–20)	15(5–20)	17(5–20)
Return	44.9	44.1	45.7	49(30–60)	53(30–60)	46(25–60)	46.3	46.1	46.5	23(10–30)	23(10–30)	23(10–30)
Other	1.6	1.5	1.7	43(15–45)	43(15–55)	42(15–45)	1.4	1.3	1.6	26(10–30)	35(10–37)	19(7–25)
Overall	100	100	100	46(25–60)	49(30–60)	44(20–60)	100	100	100	23(10–30)	23(10–30)	22(10–30)

MT = motorised transport; NMT = non-motorised transport; PT = public transport.

Walking was the transport mode for >50% of trips and more often for females than males in both cities (Table 3). The mean total travel time was shorter in Kisumu and for females in both cities.

When we grouped transport modes, NMT and informal public MT were the predominant modes, with similar transport mode shares in both cities (Table 3). Formal public transport modes were used less in Kisumu (1% vs 9.5%).

With regard to trip purposes, Kisumu had lower proportions and shorter durations of work commute trips compared to Nairobi. Main trip purposes included work, school, personal business and return home. All other trip purposes followed similar patterns in both cities (Table 3).

#### 3.2.1 Walking and cycling

The proportion of participants with a walking stage during any trip in Kisumu was half of that in Nairobi (40% vs 83%) (Table 4). Among trips including at least one walking stage, the majority (77%) of the trips included only walking in Kisumu compared to Nairobi (43%), where it was more common to walk as part of a multimodal trip. For any given trip that included at least one walking stage, walking was most likely combined with using matatus (over 60%) in both cities. The mean walking duration of trips that included matatus was double that of trips that included motorcycles in both cities.

**Table 4:** Characteristics of walking and cycling trips undertaken in Nairobi (2013) and Kisumu (2016), Kenya.

	Nairobi (*N* = 16,793)		Kisumu (*N* = 5790)	
	Walking	Cycling	Walking	Cycling
Number of participants using the mode	13,862	335	2292	130
% of participants using the mode	83	2.1	40	4
Number of trips in mode	31,559	626	4740	262
% of trips as the main mode	43	93	77	98
Number (%) accompanying modes				
Bicycle	97 (1)	..	24 (2)	..
Car	1117 (6)	3 (7)	12 (1)	00
Bus	4046 (23)	5 (12)	17 (2)	00
Matatu	11,557 (65)	26 (63)	692 (63)	2 (33)
Motorcycle	711 (4)	5 (12)	189 (17)	00
Tricycle	171 (1)	2 (5)	152 (14)	00
Taxi	00	00	6 (1)	00
Other	32 (0)	00	5 (0)	4 (66)
Walk	..	00	..	00
Median (IQR) walking time associated with other modes				
Bicycle	11(6–12)		10(7–10)	
Car	15(8–20)		7(5–6)	
Bus	17(10–20)		10(5–15)	
Matatu	17(10–20)		12(5–15)	
Motorcycle	10(6–12)		8(5–10)	
Tricycle	14(6–20)		10(5–15)	
Taxi	…		14(5–25)	
Other	19(9–26)		7(5–7)	

The proportion of participants who cycled any trip in Kisumu was double that of Nairobi (4% vs 2.1%). Where trips were cycled in both cities, they were predominantly (90%) cycled all the way. In Nairobi, cycling was sometimes combined with other modes, mainly buses, matatus, and motorcycles.

### 3.3 Correlates of trip making, transport mode usage, and travel time

#### 3.3.1 Individual trip making, trip frequency, and daily travel time

Table 5 shows the correlates of trip-making and total travel time in Nairobi and Kisumu. In Nairobi, the likelihoods of making a trip on the reference survey day were lower among females (compared to males), 05–14 and > 65 age groups (compared to 35–44 years), homemakers and people with no work (compared to those employed), and individuals living in households with motorised vehicles (compared to no vehicles). The likelihoods of making trips were higher among higher-income earners and students. This pattern was similar in Kisumu, except that there was no gender influence in trip-making in Kisumu. Similar sociodemographic factors were associated with the number of trips made in each city.

**Table 5:** Correlates of individual trip-making and daily travel time in Nairobi (2013) and Kisumu (2016), Kenya.

	AME (95%CI) Likelihood of trips		Rate Ratio (95%CI) of daily trips		%Δ (95%CI) Daily travel time	
	Nairobi	Kisumu	Nairobi	Kisumu	Nairobi	Kisumu
Female	−0.03 (−0.03, −0.02)***	0.00 (−0.03, 0.02)	0.96 (0.94, 0.98)***	0.96 (0.92, 1.01)	−9 (−11, −6)***	−7 (−13,−2)*

Age group (ref: 35–44 years)						
05–14	0.01 (−0.01, 0.02)	−0.04 (−0.09, 0.02)	0.91 (0.86, 0.97)**	0.86 (0.76, 0.97)*	−45 (−48, −42)***	−22 (−38, −3)
15–24	−0.03 (−0.04, −0.02)***	0.03 (−0.01, 0.08)	0.96 (0.93, 1)	1.07 (0.98, 1.17)	−11 (−15, −7)***	−1 (−12,11)
25–34	0.00 (0.00, 0.01)	0.06 (0.02, 0.10)**	1.03 (1, 1.06)*	1.12 (1.04, 1.21)**	−6 (−8, −3)***	−1 (−16, 17)
45–54	−0.01 (−0.03, 0.00)	0.01 (−0.05, 0.07)	1 (0.96, 1.05)	1.06 (0.94, 1.18)	1 (−3, 6)	27 (11, 45)***
55–64	−0.03 (−0.06, 0.00)	0.07 (0.00, 0.15)*	0.97 (0.89, 1.05)	1.16 (1, 1.33)*	−8 (−15, 0)	39 (9, 78)**
65+	−0.06 (−0.12, −0.01)*	0.00 (−0.10, 0.10)	0.89 (0.76, 1.04)	1.05 (0.83, 1.31)	−3 (−17, 14)	37 (5, 78)*

Income (ref: <10000KSh/month)						
10,000–30,000	0.04 (0.03, 0.05)***	0.15 (0.11, 0.19)***	1.09 (1.06, 1.12)***	1.23 (1.15, 1.33)***	13 (9, 18)***	11 (−21, 57)
>30,000	0.04 (0.03, 0.05)***	0.23 (0.18, 0.27)***	1.13 (1.09, 1.17)***	1.44 (1.3, 1.59)***	25 (20,31)***	5 (−40, 83)
No response/NA	0.00 (−0.01, 0.01)	−0.28 (−0.31, −0.24)***	1 (0.96, 1.05)	0.55 (0.51, 0.59)***	10 (6, 15)***	7 (−48, 121)

Occupation (ref: Employed)						
Homemaker	−0.10 (−0.12, −0.08)***	−0.02 (−0.07, 0.03)	0.81 (0.77, 0.85)***	1.01 (0.92, 1.11)	−21 (−29, −12)***	−26 (−34, −16)***
No work	−0.12 (−0.15, −0.10)***	−0.01 (−0.06, 0.03)	0.75 (0.71, 0.79)***	0.97 (0.89, 1.06)	−13 (−22,−2)*	−4 (−14, 6)
Student	0.02 (0.01, 0.03)***	0.04 (0.00, 0.08)	1.06 (1.01, 1.11)*	1.21 (1.1, 1.33)***	10 (4, 16)***	−3 (−21, 18)
Others	−0.04 (−0.08, 0.00)	−0.20 (−0.26, −0.14)***	0.89 (0.79, 1.01)	0.54 (0.47, 0.62)***	0 (−12,13)	−18 (−57, 57)

Household vehicle (ref: No vehicle)						
Any vehicle	0.00 (−0.01, 0.01)	−0.02 (−0.05, 0.01)	0.99 (0.95, 1.03)	1 (0.93, 1.06)	−1 (−5, 3)	−2 (−11, 8)
Motorised vehicle	−0.03 (−0.04, −0.01)***	−0.04 (−0.08, 0.00)	1.01 (0.96, 1.06)	0.94 (0.87, 1.02)	−3 (−7, 2)	−6 (−17, 7)
Inverse Mills Ratio	..	..	..	..	−19 (−44, 17)	−8 (−81, 358)
N	16,735	5767	16,735	5767	15,887	3072
R^2^ (pseudo)	..	..	0.03	0.20	0.98	0.96

AME: Average Marginal Effects; %Δ: per cent change in travel time; *** p < 0.001; ** *p* < 0.01; * *p* < 0.05.

For those with at least one trip on the reference day in Nairobi, females (compared to males), younger participants, and homemakers and those with no work (compared to those employed) had shorter total travel times. Higher-income earners and students travelled for longer durations. In Kisumu, females and homemakers travelled for shorter durations, while older people were more likely to travel for longer durations.

#### 3.3.2 Individual daily use of NMT and daily time spent in NMT

Table 6 shows the correlates of using NMT and total time spent in NMT. In Nairobi, the likelihoods of using NMT were lower among homemakers and those with no work (compared to those employed) and those earning >30,000 KES (compared to <10,000 KES) monthly. The likelihoods of NMT use were higher among those earning 10,000–30,000 KES monthly (compared to <10,000 KES) and students (compared to those employed). In Kisumu, the pattern for NMT use was similar to Nairobi, except that those living in households with vehicles were less likely to use NMT. For those who used NMT, females travelled for shorter durations in NMT than males in both cities. Higher-income earners and motorised vehicle owners were likely to travel for shorter durations in NMT modes in Nairobi but not in Kisumu.

**Table 6:** Correlates of individual use of, and daily travel time in, non-motorised transport modes in Nairobi (2013) and Kisumu (2016).

	AME (95%CI) Likelihood of NMT		%Δ (95%CI) Daily NMT time	
	Nairobi	Kisumu	Nairobi	Kisumu
Female	0.00 (−0.01, 0.01)	0.02 (−0.01, 0.05)	−6 (−8, −3)***	−18 (−26, −8)***

Age group (ref: 35–44 years)				
05–14	−0.06 (−0.10, −0.03)***	0.02 (−0.04, 0.08)	1 (−6, 8)	−4 (−19, 14)
15–24	−0.02 (−0.04, 0.00)*	0.06 (0.01, 0.10)**	−3 (−8, 1)	−17 (−37, 8)
25–34	0.02 (0.01, 0.03)***	0.06 (0.02, 0.10)**	−4 (−7, 0)*	−17 (−36, 9)
45–54	−0.04 (−0.06, −0.02)***	0.03 (−0.03, 0.09)	5 (−1,12)	13 (−9, 40)
55–64	−0.04 (−0.08, 0.00)*	0.08 (0.01, 0.16)*	−3 (−14, 8)	2 (−31, 52)
65+	−0.11 (−0.19, −0.04)**	0.05 (−0.05, 0.15)	27 (2, 57)*	52 (5, 121)*

Income (ref: <10000KSh/month)				
10,000–30,000	0.03 (0.01, 0.04)***	0.06 (0.02, 0.11)**	−11 (−14, −7)***	−30 (−45, −10)**
>30,000	−0.12 (−0.14, −0.10)***	−0.22 (−0.28, −0.15)***	−15 (−20, −9)***	14 (−54, 186)
No response/NA	−0.02 (−0.04, 0.00)*	−0.24 (−0.28, −0.21)***	4 (−1, 9)	103 (−25, 448)

Occupation (ref: Employed)				
Homemaker	−0.14 (−0.17, −0.11)***	0.02 (−0.03, 0.07)	−2 (−9, 7)	−11 (−24, 4)
No work	−0.16 (−0.19, −0.13)***	0.01 (−0.03, 0.06)	5 (−4, 15)	0 (−11, 14)
Student	0.05 (0.03, 0.07)***	0.06 (0.01, 0.10)*	−11 (−16, −6)***	−11 (−35, 21)
Others	−0.05 (−0.11, 0.02)	−0.18 (−0.23, −0.13)***	28 (11, 48)***	70 (−33,332)

Household vehicle (ref: No vehicle)				
Any vehicle	−0.01 (−0.03, 0.01)	−0.05 (−0.08, −0.02)**	10 (5, 15)***	28 (1, 62)*
Motorised vehicle	−0.20 (−0.22, −0.18)*	−0.15 (−0.19, −0.11)***	−16 (−26, −4)*	44 (−28, 185)
Inverse Mills Ratio	..	..	−22 (−41, 3)	−83 (−98, 73)
N	16,735	5767	14,012	2432
R^2^ (pseudo)	..	..	0.96	0.94

AME: Average Marginal Effects; %Δ: per cent change in travel time; *** *p* < 0.001; ** *p* < 0.01; * *p* < 0.05.

#### 3.3.3 Individual use of informal PT and time spent in informal PT

Table 7 shows the correlates of using informal PT and daily travel time in informal PT. In Nairobi, the likelihoods of using informal PT were lower among participants living in households with a vehicle (compared to no vehicle). The likelihoods were higher among students, higher-income earners, and those who were employed. The pattern for informal PT was similar in Kisumu. In addition, females were more likely to use informal PT in Kisumu. For those who used informal PT in Nairobi, total travel time in informal PT was very sensitive to sociodemographic factors. This was not the case in Kisumu, where only people without work (compared to those employed) travelled for a longer duration.

**Table 7:** Correlates of individual use of, and travel time in, informal public transport in Nairobi (2013) and Kisumu (2016).

	AME (95%CI) likelihood of informal public transport trips		%Δ (95%CI) daily informal public transport time	
	Nairobi	Kisumu	Nairobi	Kisumu
Female	0.00 (−0.01, 0.02)	0.03 (0.01, 0.05)**	−9 (−12, −6)***	−22 (−44, 8)

Age group (ref: 35–44 years)				
05–14	−0.37 (−0.40, −0.34)***	−0.16 (−0.20, −0.12)***	239 (20, 858)*	227 (−68, 3196)
15–24	−0.02 (−0.04, 0.01)	−0.01 (−0.05, 0.02)	−1 (−7, 5)	11 (−11, 39)
25–34	0.04 (0.02, 0.06)***	0.03 (0.00, 0.07)	−16 (−22, −9)***	−14 (−36, 14)
45–54	−0.08 (−0.12, −0.05)***	0.01 (−0.04, 0.06)	31 (9, 57)**	22 (−3, 53)
55–64	−0.13 (−0.19, −0.07)***	0.03 (−0.04, 0.10)	51 (11, 105)**	35 (−10,102)
65+	−0.12 (−0.23, −0.02)*	−0.10 (−0.18, −0.02)*	77 (23, 156)**	187 (−26, 1012)

Income (ref: <10000KSh/month)				
10,000–30,000	0.17 (0.15, 0.19)***	0.20 (0.15, 0.24)***	−39 (−56, −15)*	−59 (−90, 77)
>30,000	0.07 (0.05, 0.09)***	0.06 (0.00, 0.12)*	−17 (−29, −4)*	−16 (−53, 48)
No response/NA	0.00 (−0.03, 0.03)	−0.08 (−0.10, −0.05)***	8 (1, 16)*	84 (−22, 334)

Occupation (ref: Employed)				
Homemaker	−0.15 (−0.19, −0.12)***	−0.10 (−0.13, −0.07)***	57 (8, 128)*	127 (−33, 672)
No work	−0.16 (−0.19, −0.13)***	−0.04 (−0.07, 0.00)*	67 (13, 147)*	53 (2, 129)*
Student	0.14 (0.11, 0.17)***	0.04 (0.00, 0.08)	−37 (−52, −16)**	−32 (−57, 7)
Others	−0.12 (−0.20, −0.04)**	−0.09 (−0.13, −0.04)***	46 (4, 105)*	131 (−26, 618)

Household vehicle (ref: No vehicle)				
Any vehicle	−0.06 (−0.09, −0.04)***	−0.10 (−0.12, −0.07)***	23 (7, 42)**	99 (−28, 450)
Motorised vehicle	−0.10 (−0.14, −0.07)***	0.02 (−0.02, 0.05)	38 (9, 74)**	−5 (−27, 24)
Inverse Mills Ratio	..	..	−83 (−95, −49)**	−91 (−100, 204)
N	16,735	5767	7475	1204
R2 (pseudo)	..	..	0.98	0.95

AME: Average Marginal Effects; %Δ: per cent change in travel time; *** *p* < 0.001; ** *p* < 0.01; * *p* < 0.05.

#### 3.3.4 Individual use of private MT and daily travel time in private MT

Table 8 shows the correlates of using private MT and daily travel time in private MT. In Nairobi, the likelihoods of using private MT were lower among females (compared to males), most of the age groups (compared to 35–44), homemakers and those with no work (compared to those employed). The likelihood was higher among those with a higher income and those living in households with motorised vehicles. In Kisumu, the likelihoods of using private MT were lower among females (compared to males) and homemakers but higher among those earning >10,000 KES/monthly (compared to <10,000 KES) and those living in households with a vehicle (compared to no vehicle). For those who used private MT in Nairobi, the total travel time in private MT was shorter among females (compared to males) and children. No individual characteristic was found to be associated with total travel time in private MT in Kisumu. Therefore, the use of private MT in both cities is dominated by males, the wealthy and, of course, owners of private MT. This pattern is consistent with the current mobility inequity reported in Africa, where the use of a private vehicle can serve as a badge of success.

**Table 8:** Correlates of individual use of, and daily travel time in, private motorised transport in Nairobi (2013) and Kisumu (2016), Kenya.

	AME (95%CI) Likelihood of private car trips		%Δ (95%CI) Daily private car travel time	
	Nairobi	Kisumu	Nairobi	Kisumu
Female	−0.02 (−0.03, −0.02)***	−0.03 (−0.04, −0.01)***	−16 (−22, −9)***	6 (−24, 49)

Age group (ref: 35–44 years)				
05–14	−0.08 (−0.11, −0.06)***	−0.05 (−0.07, −0.03)***	−50 (−66, −26)***	−24 (−70, 91)
15–24	−0.08 (−0.09, −0.06)***	−0.02 (−0.04, 0.01)	−16 (−38, 13)	37 (−2, 91)
25–34	−0.03 (−0.04, −0.02)***	0.01 (−0.01, 0.02)	−6 (−14, 3)	8 (−12,31)
45–54	0.03 (0.01, 0.05)***	−0.01 (−0.04, 0.01)	−3 (−11, 7)	20 (−11, 62)
55–64	0.04 (0.01, 0.08)*	0.01 (−0.03, 0.05)	−10 (−22, 4)	5 (−28, 53)
65+	0.01 (−0.05, 0.07)	−0.04 (−0.09, 0.01)	−21 (−39, 3)	−8 (−74, 216)

Income (ref: <10000KSh/month)				
10,000–30,000	0.01 (0.00, 0.02)	0.04 (0.01, 0.06)**	9 (−4, 24)	9 (−27, 61)
>30,000	0.18 (0.16, 0.20)***	0.27 (0.21, 0.33)***	16 (−22, 71)	10 (−71, 311)
No response/NA	−0.01 (−0.02, 0.01)	−0.03 (−0.04, −0.01)***	16 (−3, 40)	35 (−19, 125)

Occupation (ref: Employed)				
Homemaker	−0.06 (−0.08, −0.04)***	−0.04 (−0.06, −0.02)***	−26 (−47, 2)	−36 (−71, 46)
No work	−0.04 (−0.07, −0.02)***	−0.02 (−0.04, 0.01)	15 (−21, 68)	−17 (−47, 29)
Student	0.03 (0.00, 0.05)*	−0.01 (−0.04, 0.01)	−16 (−34, 6)	−31 (−54, 5)
Others	−0.01 (−0.06, 0.04)	0.00 (−0.04, 0.03)	15 (−25, 78)	−12 (−44, 39)

Household vehicle (ref: No vehicle)				
Any vehicle	0.00 (−0.02, 0.02)	0.06 (0.04, 0.08)***	−15 (−32, 6)	18 (−50, 176)
Motorised vehicle	0.18 (0.16, 0.20)***	0.06 (0.04, 0.07)***	41 (−21, 150)	−29 (−63, 37)
Inverse Mills Ratio	..	..	1 (−40, 69)	−28 (−85, 244)
N	16,735	5767	2314	441
R2 (pseudo)	..	..	0.98	0.95

AME: Average Marginal Effects; %Δ: per cent change in travel time; *** *p* < 0.001; ** *p* < 0.01; * *p* < 0.05.

### 3.4 Comparing data sources

The Nairobi (2013) and Kisumu (2016) travel surveys, and the World Bank (2013) (Gulyani et al., 2013) baseline surveys, showed similarities and substantial differences in terms of work and school commute characteristics (Table 9). All three surveys imply that walking and cycling rates are higher in Kisumu than in Nairobi, while the use of cars and matatus is higher in Nairobi than in Kisumu, although the mode shares are different. The World Bank (2013) baseline survey reported that the predominant work and school commute modes (∼90%) in both cities are walking and the use of matatus; Nairobi (2013) reported the same predominant commute modes accounting for 67% of trips, while Kisumu (2016) reported different predominant commute modes (walking and use of motorcycles) accounting for 68% of trips (Table 12). For Nairobi, matatu and bicycle shares are comparable, while walking, bus, and car shares are widely different between the two surveys. For Kisumu, bus and bicycle shares are comparable, while the other modes are widely different between the two surveys.

**Table 9:** Comparing work and school commute transport mode shares and travel times from the Nairobi (2013) and Kisumu (2016) travel surveys and the World Bank (2013) baseline survey.

		Nairobi			Kisumu	
	Share (%)	Median (IQR)	Mean (SD)	Share (%)	Median (IQR)	Mean (SD)
World Bank data	*N* = 1940			*N* = 1535		
Walk	52.4	15(5–20)	17(1)	59.8	15(10–30)	20(1)
Bicycle	1.7	10(4–20)	15(2)	3.8	20(15–30)	22(2)
Bicycle taxi	0.5	7(7–20)	12(3)	4.6	15(7–20)	15(1)
Car	4	30(15–45)	76(36)	1.9	10(10–20)	24(7)
Matatu	37.5	30(20–60)	49(3)	26.8	30(20–40)	48(5)
Taxi shared	0.1	10(10−10)	10(0)		..	..
Taxi vehicle	0.1	10(10–10)	10(0)	0.1	30(30−30)	20(5)
Bus	0.4	10(10–60)	33(14)	0.8	12(10–60)	97(38)
Other	3.3	7(0−20)	26(9)	2.2	10(7–25)	32(14)
Overall	100	20(10–30)	31(2)	100	20(10–30)	28(1)
Travel survey data	N = 15,169			*N* = 2076		
Walk	37.7	30(15–30)	31(1)	60.3	15(10–30)	21(1)
Bicycle	1.6	30(20–60)	42(2)	4.5	18(10–30)	19(1)
Motorcycle	3.6	30(25–45)	38(1)	17.2	15(10–20)	18(1)
Tricycle	1	40(30–60)	46(2)	2.4	25(18–35)	28(2)
Car	13.1	45(30–60)	52(1)	1.5	33(25–50)	59(25)
Matatu	28.8	50(30–60)	57(1)	7.4	35(25–45)	38(2)
Bus	12.7	50(30–60)	55(1)	6.1	20(10–25)	25(4)
Other	1.5	40(30–60)	50(3)	0.6	24(10–95)	72(37)
Overall	100	30(30–60)	45(0)	100	20(10–30)	23(1)

In terms of commute time by mode, the World Bank data showed that apart from cars, commute times for all modes were higher in Kisumu than in Nairobi. On the contrary, when using the Nairobi (2013) data and the Kisumu (2016) data, commute times were higher for all modes in Nairobi than in Kisumu. Although commute times were generally lower in the World Bank data, the time difference was more pronounced with the Nairobi (2013) data than with the Kisumu (2016) data.

Overall the Kisumu travel survey closely matches the World Bank estimates in terms of commute duration and mode shares for NMT but not other modes. Similarly, the Nairobi travel survey closely matches the World Bank estimates in terms of NMT shares but significantly deviates when it comes to commute durations.

## 4 Discussion

### 4.1 Main findings

This is one of the very few studies addressing the literature gap on travel behaviour in African cities. It describes and compares travel behaviour characteristics and correlates between Nairobi, the capital city of Kenya, and Kisumu, the third-largest city in Kenya. We found a higher rate of no reported trips in Kisumu compared with Nairobi (47% vs 5%), with double the average number of trips per survey participant in Nairobi (2.3; IQR:2–2 vs 1.2; IQR:0–2). The mean trip duration and daily travel time in Nairobi were double those in Kisumu. Walking was the most common transport mode, followed by matatus in both cities. Most walking (53%) was undertaken in combination with other modes in Nairobi, whereas walking was the sole transport mode in most walking trips in Kisumu (77%).

Gender, income, occupation, and household vehicle ownership were variedly associated with individual trip making; use of NMT, informal PT, and private MT; and individual daily travel times in different transport modes. For example, in Nairobi, females, compared to males, were less likely to make trips (AME = −0.03; 95%CI = -0.03 to −0.02), and those who made trips travelled for shorter times (% difference = −9; 95%CI = −11 to −6). In Kisumu, gender was not associated with trip making; for those who travelled, females travelled for shorter times than males (%difference = −7; 95%CI = −13 to −2). Living in households earning >10,000 KES ($100) per month was associated with a higher probability of trip-making and longer daily travel times in both cities. Females and people living in households with higher monthly incomes travelled for shorter durations in NMT modes and had higher chances of using informal PT in both cities.

Other relevant studies in the literature focused on testing advanced travel survey designs (Behrens and Behrens, 2004), analysis methods (Gebeyehu and Takano, 2007), and the use of public transport (Abane, 2011), with none of these studies using representative data. Salon et al. recently conducted two analyses on travel patterns from representative surveys in Kenyan cities: one based on a 2004 travel survey in Nairobi (Salon and Aligula, 2012) and the other based on the 2013 World Bank data on commuting in major Kenyan cities (Salon and Gulyani, 2019). However, the 2004 data are likely outdated, while the 2013 data reports only commuting. This study, therefore, provides comprehensive and relatively timely findings that respond to a critical gap in the literature and inform transport policies.

### 4.2 Limitations

Despite filling an important gap in the literature, our study does have limitations, most of which are related to the data sources. Firstly, because the datasets originated from different organisations, collected at different times and for slightly different purposes, with possible contextual adaptations, there are likely inherent differences between the data sources. To account for differences resulting from data types, we explored our two main datasets and compared them with other available data. In Nairobi, four other representative surveys with travel-related questions have been conducted since 2004. One of these surveys was conducted by the Japan International Cooperation Agency (JICA) in 2004; the second survey by the Kenya Institute for Public Policy Research and Analysis (KIPPRA) in 2004, the third survey in 2010 by the African Centre of Excellence for Studies in Public and Non-Motorised Transport (ACET), and the fourth survey conducted by the World Bank in 2013. Many parameters have been found to substantially differ among these surveys, with JICA surveys reporting higher car mode share, fewer walkers and longer travel durations compared to all the other surveys (Salon and Gulyani, 2019). There is consistency in the travel duration of motorised travel and the fraction of walking time in these surveys (Salon and Gulyani, 2019). In Kisumu, apart from the ITDP data, only the World Bank collected data with travel variables, but these two datasets have not been previously compared. Our triangulation of the two main datasets, with a third dataset that covered both settings, provided a lens through which our findings were interpreted. Travel surveys are prone to recall bias and ‘soft refusal’ (participants reporting no travel to avoid filling out the rest of the questionnaires), as surveys entail recalling multiple travel activities, estimating travel times, and filling out relatively lengthy questionnaires. The reports on the administration of both surveys provide confidence in the steps taken to ensure correct data. However, the trip rate in Kisumu is low and warrants further investigation.

### 4.3 Interpretation of findings

#### 4.3.1 Trip-making and daily travel time

The two cities have very contrasting rates of reporting no trips on the survey day (5% Nairobi vs 47% Kisumu). These values deviate substantially from the admitted 8 to 12% of the population that will be captured as immobile at some point (Madre et al., 2007), even if this global figure varies substantially across age groups, zone of residence, and days of the week. One also notes that the trip rates in Kisumu are similar for men and women. A possible explanation for the high no-trip rates in Kisumu is ‘soft refusal’ or participants omitting shorter trips if interviewers did not skilfully prompt their inclusion (Madre et al., 2007). On the other hand, the very high reported participation rates in Nairobi could signal that people without trips were somehow excluded. The case for possible overestimation of travel behaviour characteristics in Nairobi is strengthened by the long travel times reported in the city.

However, the large observed differences strongly suggest considerable differences in participation and travel times between two major cities in the same country.

Our findings that females were less likely to make trips, and when they travelled, they travelled for shorter times than males support recent claims on gender inequity in travel in Africa (Foley et al., 2021). In addition, mobility increases with household income, highlighting a possible financial barrier to motorised transport for many. Notwithstanding the general trend, being female did not seem as much of an impedance to travel in Kisumu than Nairobi. A link could not yet be made between the inclusive mobility observed in Kisumu and the recent changes in transport policies supported by NGOs such as the ITDP and the city's campaign to host major events such as the Africities summit in 2022.

#### 4.3.2 NMT

The high use of NMT modes is expected in most African cities, and cities with higher NMT use such as Kisumu have taken steps to preserve NMT in their mobility plans with actions including education and advocacy for NMT, the introduction of car-free days, and improving private car parking facilities to create space for public transport and NMT use (Institute for Transportation and Development Policy, 2020). Participants aged 45 years and above and those living in households with monthly incomes above 30,000 KES or with motorised vehicles were less likely to use NMT modes in both cities. This observation is consistent with the expectation that people earn more, purchase vehicles and abandon NMT modes as they age. In terms of physical activity accumulated from the use of NMT modes, over half of the participants engaged in moderate levels of physical activity in the form of walking and cycling for >30 min on the survey day. The importance of travel as a source of physical activity in lower-income countries is corroborated by Strain et al. in their work on domain-specific physical activity in 104 countries (Strain et al., 2020b). Although most of the NMT comes from walking, cycling is significant in Kisumu, ranking among the top global cycling cities, albeit dominated by men (Goel et al., 2022a; Goel et al., 2022b). The difference in cycling rates between Nairobi and Kisumu could be a result of multiple factors, including historical and built environmental factors. For example, the built environment is denser in Nairobi because of the city's large population and the city's role as the capital.

Our regression analysis also showed some unexpected results. The positive coefficients between daily trips and no work and the negative correlation between ownership of motorised vehicles and the likelihood of trips are intriguing results that warrant further exploration. One possible explanation for the positive correlation between daily trips and no work could be that individuals who are not employed have more flexible schedules and are, therefore, more likely to engage in non-work-related trips and job searches. The negative correlation between ownership of motorised vehicles and the likelihood of trips could be explained by the fact that individuals may own cars for luxury and not for essential travel. The results also showed that wealthier people are more likely to use informal public transport, probably because this study's wealthy bracket (≥10000KSH) still includes most people who would typically use informal PT. However, it is important to note that these explanations are speculative and further research is needed to understand the underlying factors that fully contribute to these findings.

An ad-hoc analysis of the perceptions of the environment and opinions on improving travel modes in Kisumu shed more light on our findings. Most participants report acceptable neighbourhood public transport, low road traffic congestion, high crime rates, inadequate footpaths, and poor street lighting. The observed low mobility means that the high crime rates, inadequate footpaths and poor lighting could be strong barriers to mobility. The perception of a good transport environment in terms of access and low traffic does not seem to imply high mobility. Page et al. (Page et al., 2010) reported how the perception of a physical environment relates differently to physical activity behaviours, with young girls but not boys engaging in outdoor plays when they perceived less risk in road traffic accidents, while boys but not girls increased active commuting to school if they perceived easy accessibility. In addition, as expected, participants in the Kisumu survey considered the safety of different modes as a key strategy for improving the usage of transport modes.

### 4.4 Implications of findings

The high proportion of participants with no trips in Kisumu needs further exploration, especially using improved data, but highlights the importance of consistent study designs with attempts to overcome soft refusals in travel surveys. From the lenses of income, gender, adolescence, and the elderly, it was more inclusive and equitable when people were mobile. The low proportions of people with trips raise concerns about potential immobility in smaller towns and cities that make up most of Africa's urban areas. Most African cities are slightly smaller than Kisumu in size and stage of urban development, with over 97% of African urban areas having fewer than 300,000 inhabitants (OECD, 2020). Much of Africa's projected rapid urban growth will occur in these small and medium-sized cities (OECD, 2020).

The observed growth in motorcycles equally concerns from two perspectives. Firstly, motorcycle trips seem to pull from NMT and other public transport modes such as matatus. This reduces opportunities for active travel and obscures the demand for further development of walking, cycling and public transport infrastructure. Secondly, motorcycle riders and passengers typically face high injury risks and pose a high risk to pedestrians.

Kisumu's relatively higher cycling uptake indicates the possible success of promoting cycling use in similar settings. To encourage and consolidate high cycling levels, there is a need for investments to reduce danger and increase the safety of the mode, as reflected by the participants' opinions on improving the safety of the different transport modes. Efforts to increase cycling must also consider the competition induced by the rapid increase in motorcycling.

The data challenges encountered in this study, including limited datasets, gross disparities in estimates, and omission of key variables, highlight known problems with data that inform travel behaviour. Travel data from cities worldwide have often posed challenges that impede the comparison and monitoring of travel patterns. These challenges include the frequent unclear definition of trips, inconsistent inclusion of short trips, poor selection of travel days to adequately capture weekly and seasonal variation, the granularity of travel time estimates and the designation of trip modes. Efforts to harmonise and triangulate travel data are necessary to paint more complete pictures of travel behaviour and the evolution of travel patterns.

## 5 Conclusion

Our findings show that the proportion of individuals travelling is very high in Nairobi and very low in Kisumu; also, the travel durations are longer in Nairobi and shorter in Kisumu. Higher household income is associated with more travel in both cities, while the female gender is associated with less travel in Nairobi and shorter travel times in both cities. The safety of different transport modes is considered a key strategy for improving mode usage in Kisumu. The marked differences in travel behaviour characteristics and correlates between cities in the same country indicate setting-dependent correlates of travel behaviour. This suggests the need for more sub-national data collection to build a more nuanced understanding of travel behaviour profiles, patterns, and drivers in African cities.

## Funding

This research was funded by the 10.13039/501100000272National Institute for Health Research (NIHR) (GHR: 16/137/34) using UK aid from the UK Government to support Global Health Research. The views expressed in this publication are those of the authors and not necessarily those of the NIHR or the UK Department of Health and Social Care. LT and JW were supported with funding from the 10.13039/501100000781European Research Council (ERC) under the Horizon 2020 Research and Innovation Programme (grant agreement No 817754).

## Data Availability

The authors do not have permission to share data.
